# Mental health system at the community level in Korea: development, recent reforms and challenges

**DOI:** 10.1186/s13033-019-0266-y

**Published:** 2019-02-13

**Authors:** Yong-Chang Heo, Sang Kyoung Kahng, Sungyong Kim

**Affiliations:** 1 Department of Social Welfare, Keimyung University, 331-2, College of Social Science Building, 1095, Dalgubeol-daero, Dalseo-gu, Daegu, Republic of Korea; 2 Department of Social Welfare, Seoul National University, 16-651, College of Social Science Building, 1, Gwanak-ro, Gwanak-gu, Seoul, Republic of Korea; 3 Korean Research Center for Guardianship and Trusts (KCGAT), 222 Wangsimni-ro, Seongdong-gu, Seoul, Republic of Korea; 4 Institute of Social Welfare, Seoul National University, 220-551, 1, Gwanak-ro, Gwanak-gu, Seoul, Republic of Korea

**Keywords:** Mental Health Act, Mental health reforms, Community care, Continuing challenges

## Abstract

Since the introduction of the Mental Health Act (MHA) in 1995, mental health services have expanded at the community level in Korea. While community facilities for mental health have grown considerably, large numbers of people with mental disorders are accommodated as before in private mental hospitals. Korea needs to reduce the level of dependence on long-term treatments in hospitals and expand coverage of services for the people with mental illness (PMI) to all in local communities. To achieve this objective, the significant legislative changes were made through the amendment of the MHA. The completely revised act indicates that the Korean government seeks a harmonized balance between inpatient care and outpatient care by declaring the necessity of various welfare services to ensure human rights of the PMI. Particularly, mental health system furthers to provide comprehensive services for the majority of community population to monitor risk factors of mental disorders as well as for the PMI. In this sense, the Korean government could refer to the British case of “Improving Access to Psychological Therapies” as suggested by an OECD investigation team. Achieving the goal calls for both a deliberate realignment of existing services and additional resources in line with legislative reforms. Further public efforts should be made in collaboration with medical institutions and private service providers to realize the valuable goals pursued by the amended act.

## Background

Since the introduction of Mental Health Act (MHA) in 1995, mental health services have expanded at the community level in Korea. According to the Ministry of Health and Welfare [1], there has been an approximately fivefold increase in the number of facilities for mental health rehabilitation from 66 in 2001 to 333 in 2015, and Mental Health Welfare Centers (MHWCs) from 46 in 2001 to 253 in 2015 over the last 15 years. While community facilities for mental health have grown considerably, large numbers of people with mental disorders are accommodated as before in private mental hospitals. The dependence on mental health hospitals, for example, is well demonstrated in data showing that the numbers of beds in private mental hospitals (76,629) are ten times those of the accommodated people in mental health rehabilitation facilities (7041). These figures imply that despite the quantitative growth in the number of facilities for mental health services in communities, the Korean mental health system still largely relies on inpatient care in mental hospitals despite the increasing number of mental disorders. Additionally, an Organisation for Economic Co-operation and Development (OECD) investigation team suggested that, given the high level of stress across major demographic groups, community mental health system should be comprehensively reformed to diagnose, manage, and treat mental disorders for the general population [2]. Significantly affected by continuing critiques of institutionalization, the Korean government intends to enhance community-based care services, not only for the people with mental illness (PMI) but also the public in general following the suggestions from an OECD investigation team. These changes in policy orientation pose two significant implications. First, the reforms will attempt to curb hospitalized treatments as much as possible by reinforcing admissions reviews. They also serve to promote the human right of the PMI against involuntary hospitalization. Second, the reforms intend to extend the coverage of public support to the majority of the population. These reforms can be understood when considering that a community mental health system should cope with increasing risk factors e.g. high suicide or depression rates among the population (e.g. see [3, 4] for the review of suicide and depression issues in Korea). Consequently, the MHA was completely revised and newly came into force in 2017 seeking to improve the mental health of the public and support the PMI. However, most MHWCs as primary bases for community care in mental health have considerable difficulties in meeting the rising level of demand for services, as many past reforms were pushed into practice without essential investments in financial and/or human resources. Given the lack of further public inputs, it is unlikely that the new act will immediately bring about positive shifts toward community-based care.

Given these uncertain circumstances, this paper aims to examine recent reforms in the community mental health system and controversial issues related to these reforms in Korea. A case study, broadly in a comparative context, as conducted here can help other middle-income and low-income countries to draw practical implications for better mental health systems.

## Case presentation

Significant legislation was enacted in the form of the 1995 MHA for the Korean mental health system. Prior to the enactment of the MHA, the Korean mental health system practically leaned toward long-term hospitalization, where most PMI were accommodated and treated in psychiatric hospitals [5]. The act aimed not only to legalize the process of involuntary admission to psychiatric hospitals but also to reinforce the provision of mental health services at the community level in general [6]. Therefore, the MHA can be regarded as the first act which regulated the process of treatment for mental disorders and declared the importance of community mental health services in Korea. Additionally, the act intended to establish a well-organized community mental health system for prevention, treatment, nursing and rehabilitation [7]. To achieve this goal, community mental health services began to be institutionally organized by the establishment of centers for mental health promotion, currently designated as Mental Health Welfare Center, after the MHA was passed. Four centers started to provide services for the mentally disabled in 1998 as a pilot project [8]. It was not until 2005 that the pilot project was terminated and the centers for mental health promotion were officially operated across the country [9]. When the centers were founded, their major roles were to provide case management, counseling and group programs for local residents who suffered from severe and persistent mental illnesses (SPMI). Regarding qualifications for service providers, the state brought in a certification system to label those as ‘mental health professionals’ in 1997. In addition to psychiatrists, mental health professionals included mental health social workers, mental health nurses, and clinical counselors. Community mental health services continued to be developed in 2007, when several new programs were launched. Children and juveniles began to be subjects of mental health service programs with checkups on mental health for the juveniles beginning at the age of sixteen. Other programs were also provided to treat and prevent alcohol dependence in accordance with Blue Bird Plan 2010 initiated by the central government. With regard to subsidizing programs and consumer choices, vouchers for total care services were introduced and assigned to the PMI who resided in the relevant local community. With these new programs, the coverage of community mental health services was extended to other demographic groups beyond adults with mental illness and the process by which subsidies were allocated shifted to supporting consumers rather than providers in order to give preferences to consumers [8].

In 2016, the MHA was completely revised and newly entitled as the “Act on the improvement of mental health and the support for welfare services for mental patients” (AMSW). The AMSW was affected by the 2016 Mental Health Project Plan, designed by the MOHW, stressing the importance of mental health for the entire population and to help people who suffered from SPMI to become established in their local community. The act was also in line with the latest recommendations by an OECD investigation team, which suggested that mental health policies in Korea require further support for the public as well as patients. According to Article 1, the AMSW aims to contribute to improving public mental health and the lives of those who are PMI by providing medical treatments and required services and by promoting a friendly system for these PMI. To pursue the core goals, the central government and local governments should assume shared responsibility to take preventive measures to improve public mental health and formulate comprehensive policies for the protection of human rights for the PMI and their families while also providing supportive services (Article 4). The AMSW seeks not only human rights of people who suffered from SPMI but also to expand public investment in mental healthcare promotion. Regarding the rights of patients, the act enhances admissions reviews by requiring checks by two independent psychiatrists and implements a 2-week temporary hospitalization stay for a precise diagnosis (Article 43). Prior to the act, patients could be hospitalized by their families or guardians upon a diagnosis by a single psychiatrist, which violated self-determination of patients given that the constitutional court adjudicated regarding this clause. Additionally, for broader coverage, the AMSW intends to offer comprehensive services for the PMI by defining key service categories, such as rehabilitation, employment, lifelong education and leisure, for wide-ranging support at the community level. In addition to those at high risk, the act extends the scope of mental health services to the general population to detect potential risks and prevent mental disorders in advance.

**Fig. 1:**
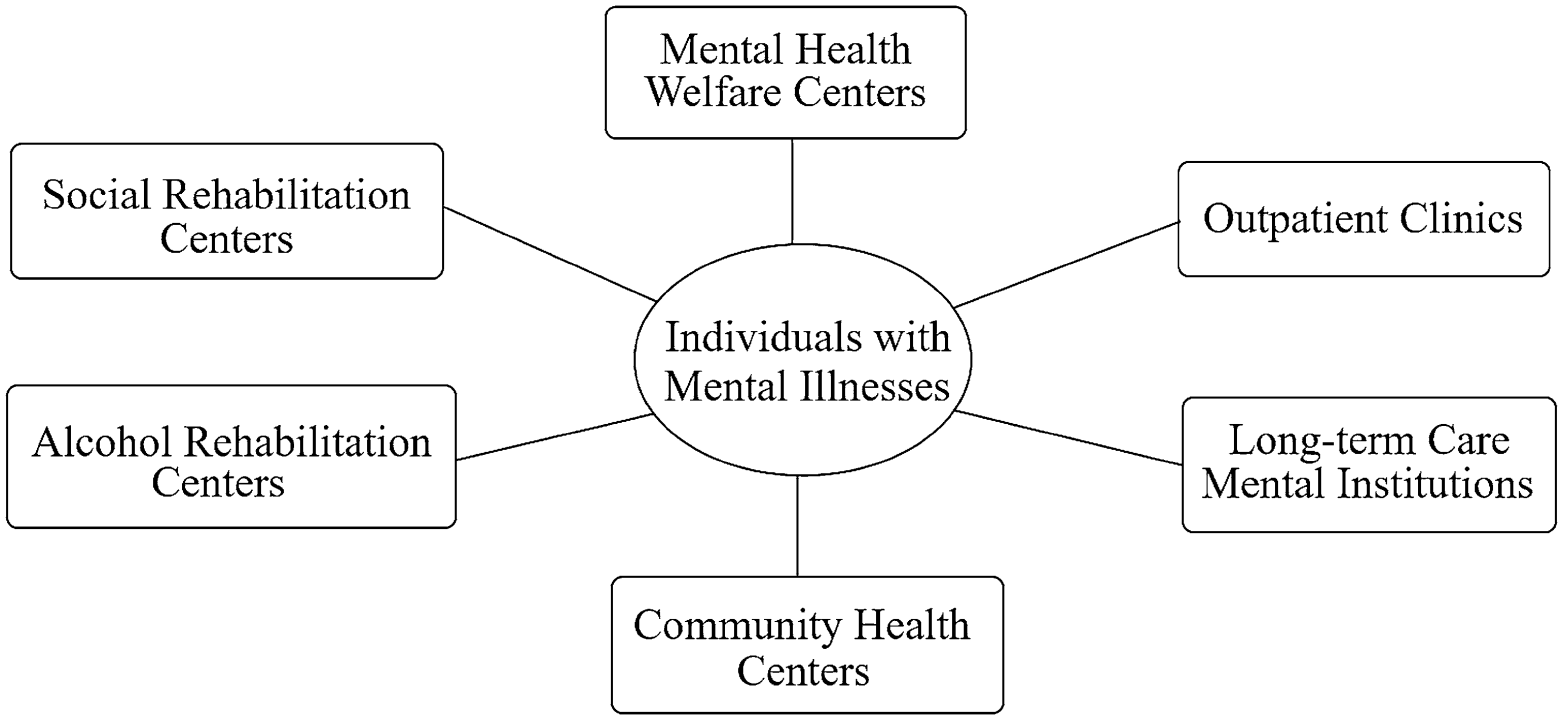
Mental health system at the community level

As the Korean government organized mental health services, a public mental health system has been steadily established over the last two decades. The current mental health system, as seen in  Fig. 1, consists essentially of medical institutions and service providers for rehabilitation and settlement in the local community. Particularly, MHWCs play a pivotal role in delivering essential services at the community level. In terms of public expenditures in the community mental health sector, approximately 85% of the total expenditure was given to MHWCs to subsidize mental health programs by the central government in 2014 [10]. In 2015, 224 MHWCs in total were operated across the country. Their key programs are as follows: suicide prevention, mental health promotion mainly for the young and elderly, addiction management, programs for North Korean defectors and immigrants, and case management for the PMI [11]. While most programs offered by the centers are designed for the PMI to support treatment and rehabilitation, some are extended to other segments of the population to prevent mental disorders in advance.

## Discussion and conclusions

Despite these developments in community mental health services, critical issues remained unresolved. Most of all, the current mental health system is still framed by the hospitalization model as opposed to taking a community-based approach. There are 1449 mental health hospitals, accounting for approximately 68% of all institutions and facilities for mental health [11]. These figures show that many people who have mental disorders, in general, are assumed to be hospitalized and receive medical treatment. Additionally, public medical insurance scarcely imposes significant disadvantages on long-term patients treated in mental hospitals. As a result, many patients become long-term residents at these facilities and lose their will to return to their own communities. The provision of mental health services also is neither sufficient nor well organized at the community level for the entire population and nor are these services organized for the PMI. For instance, the numbers of rehabilitation facilities remain insufficient to accommodate the PMI, and they are unevenly distributed across countries. According to MOHW [1], among the seven metropolitan cities in Korea, 59% of all boroughs, labeled as ‘*gu*’ in these cities in Korea do not operate a single rehabilitation facility. This figure represents the wide disparities in the regions and types of service provider, and this lack of rehabilitation facilities contributes to treated patients involuntarily staying at mental hospitals owing to unaffordable housing situation.

Although the recent AMSW stressed the importance of comprehensive services for the PMI, most service providers are faced with considerable difficulties to fulfill their legal duties in the mental health system. Hong et al. [12] classified the problems of the existing system with the following categories of services in the act. First, MHWCs are overloaded with increased numbers of mental health promotion programs and thus underperform in case management given the lack of human and financial resources. Second, there are too few housing facilities, such as long-term care mental institutions and group homes are not enough to accommodate the PMI when they leave their families or hospitals. Third, job training services are currently provided by only ten service providers across country, most of which are located in the Seoul metropolitan area. These problems indicate that most daily services for the PMI are generally underprovided due to low levels of concern in the service infrastructure. These problems largely stem from the origin of the mental health service system in Korea. At the early phase of development, most programs served limited numbers of local residents with special mental needs and not the entire community. It was not until recently that mental health services were extended to other people without SPMI. Hence, despite recent reforms, the public sector and private service providers share common tasks to ensure that the key goals of the revised act can be achieved.

Since the legislation of the MHA, the Korean government has striven to lay the foundation of a community-centered system in the mental health sector over the last two decades. In particular, MHWCs and related service facilities have played key roles to provide mental health services at the community level, but they face challenging tasks to cope with various issues related to the public as well as those with SPMI. The recent amendment of the MHA was notable progress which effectively handled the growing level of demand for mental health services. The former MHA was reformed to break the mental health system from hospitalized treatment and expand the coverage of community services to the general population. Notwithstanding the ambitious goals, concrete plans including additional resources were not presented to accomplish the goals. Unless additional investments in the service infrastructure are entailed as soon as possible, many communities may face difficulty in meeting the rising demand for community mental health services under the amended act.

Although remarkable efforts have been undertaken to tackle global health inequalities, efforts to improve mental disorders has a long way to go around the world, especially in low-income countries [13]. Among rapidly developing countries, Korean society created a substantially improved mental health system in which most services were designed for the PMI. It was found that this approach laid down essential services which were indispensable to local residents with SPMI and promoted their welfare to a considerable degree. On the other hand, Korea needs to reduce the level of dependence on long-term treatments in hospitals and expand coverage of services for the PMI to all in local communities. As the initial MHA failed to achieve this objective, significant legislative changes were made through the amendment of the MHA. The completely revised act indicates that the Korean government seeks a harmonized balance between inpatient care and outpatient care by declaring the necessity of various welfare services to ensure human rights of the PMI. Particularly, mental health system furthers to provide comprehensive services for the majority of community population to monitor risk factors of mental disorders as well as for the PMI. In this sense, the Korean government could refer to the British case of “Improving Access to Psychological Therapies” as suggested by an OECD investigation team. Achieving the goal calls for both a deliberate realignment of existing services and additional resources in line with legislative reforms. Further public efforts should be made in collaboration with medical institutions and private service providers to realize the valuable goals pursued by the amended act.

## Abbreviations

MHA: Mental Health Act
MOHW: Ministry of Health and Welfare
MHWC: Mental Health Welfare Center
SPMI: severe and persistent mental illnesses
AMSW: Act on the improvement of mental health and the support for welfare services for mental patients
OECD: Organisation for Economic Co-operation and Development
